# Simulation and Analysis of the Effects of Land Use and Climate Change on Carbon Dynamics in the Wuhan City Circle Area

**DOI:** 10.3390/ijerph182111617

**Published:** 2021-11-04

**Authors:** Chao Liu, Yuan Liang, Yajin Zhao, Shuangshuang Liu, Chunbo Huang

**Affiliations:** 1 Research Center for Spatial Planning and Human-Environment System Simulation, School of Geography and Information Engineering, China University of Geosciences, Wuhan 430078, China; liuchao@cug.edu.cn (C.L.); liushuangshuang@cug.edu.cn (S.L.); 2 School of Arts and Communication, China University of Geosciences, Wuhan 430078, China; liangyuan612@cug.edu.cn; 3 Dalian Customs District P.R. China, Dalian 116000, China; viticulture2014318@gmail.com

**Keywords:** landscape planning, carbon sequestration, NPP (Carnegie–Ames–Stanford Approach), ecological restoration, CASA model, urbanization

## Abstract

In a climate and land use change context, the sequestration of atmospheric carbon in urban agglomeration is key to achieving carbon emission and neutrality targets. It is thus critical to understand how various climate and land use changes impact overall carbon sequestration in large-scale city circle areas. As the largest urban agglomeration in central China, carbon dynamics in the Wuhan City Circle area have been deeply affected by rapid urbanization and climate change in the past two decades. Here, we applied monthly climate data, spatially explicit land use maps, NDVI (Normalized Difference Vegetation Index) images and the CASA (Carnegie–Ames–Stanford Approach) model to estimate the spatial and temporal changes of carbon dynamics in the Wuhan City Circle area from 2000 to 2015. We designed six different scenarios to analyze the effects of climate change and land use change on carbon dynamics. Our simulation of NPP (Net Primary Productivity) increased from 522.63 gC × m^−2^ to 615.82 gC × m^−2^ in the Wuhan City Circle area during 2000–2015. Climate change and land use change contributed to total carbon sequestration by −73.3 × 10^10^ gC and 480 × 10^10^ gC, respectively. Both precipitation and temperature had a negative effect on carbon sequestration, while radiation had a positive effect. In addition, the positive effect on carbon sequestration from afforestation was almost equal to the negative effect from urbanization between 2000 and 2015. Importantly, these findings highlight the possibility of carrying out both rapid urbanization and ecological restoration simultaneously.

## 1. Introduction

With the Chinese government putting forward the goals of peak carbon emissions in 2030 and carbon neutrality in 2060, regional research on carbon sequestration and emissions reduction is becoming more and more important [1]. Climate change and human-induced land use change are two important effects on the global and regional carbon cycle [2,3]. The main reason for this is that they can significantly change regional biodiversity, landscape patterns, ecosystem functions and services [4,5]. Decoupling the impacts of climate change and human-induced land use change on carbon sequestration in regional ecosystem is a feasible way to reveal the influence of climate change and land use change on ecosystem services [6]. For example, some previous studies [5,7,8] reported that urban expansion and the implementation of green infrastructure projects (e.g., afforestation, reforestation) can occur simultaneously in the same region. Therefore, the superposition of positive and negative effects will lead to more complex and difficult understanding of carbon dynamics under different land use conditions [9]. Meanwhile, the lack of such information can also lead to misinterpretation of the linkage between land use change and climate change [10]. Consequently, it is important to quantitatively evaluate and analyze the magnitude and direction of the respective and combined effects of various changes in climate and land use on carbon dynamics.

Net primary productivity (NPP) is one of the most important indicators for revealing ecosystem carbon dynamics [11]. Accurate and rapid estimation of NPP at regional scales is of great significance for assessing the regional ecosystem carrying capacity and rational utilization of natural resources [12]. Because it is impossible to directly and comprehensively measure ecosystem productivity on a regional or global scale, using models to estimate and simulate carbon dynamics in terrestrial areas has become an important and widely accepted research method [13,14,15]. The Carnegie–Ames–Stanford Approach (CASA) model, which is a process-based light use efficiency model [16], has been widely used to simulate terrestrial carbon dynamics under climate change or land use change. Based on some previous studies [16,17,18,19,20], the CASA model has been proved to be well applied to the simulation of NPP in various regions in China. These studies also provide some fundamental support for further land use policies towards carbon neutrality at the regional scale in China. To the best of our knowledge, there is no study that has used the CASA model to estimate the carbon dynamics of the Wuhan City Circle area in China.

The Wuhan City Circle area is an important strategic fulcrum of the central rise plan in China [21]. This region has great opportunities to develop its economy and improve its urban structure and layout. However, the pressure of environmental protection and carbon emission reduction is also an important factor in maintaining sustainable development in this region [22]. With rapid development and urbanization, the effects of land use change and climate change on carbon dynamics in the Wuhan City Circle area have become more obvious in the past two decades [23]. In this study, we used the CASA model to estimate the NPP of the Wuhan City Circle area and explored the spatiotemporal dynamics of carbon from 2000 to 2015. Six different scenarios were estimated in order to analyze the relative roles of various climate and land use changes on carbon dynamics. Based on this study, we were able to provide a theoretical foundation for further ecological construction in the Wuhan City Circle area and contribute to achieving carbon neutrality at the regional scale by strengthening land use policies. The specific objectives were as follows: firstly, to simulate and characterize the spatiotemporal variation in total carbon sequestration in the Wuhan City Circle area from 2000–2015; secondly, to evaluate the overall effect of climate and land use changes on carbon dynamics in the Wuhan City Circle area; and finally, to reveal the relative impacts of various climate and land use changes on the carbon dynamics of the Wuhan City Circle area.

## 2. Materials and Methods

### 2.1. Study Area

**Figure 1:**
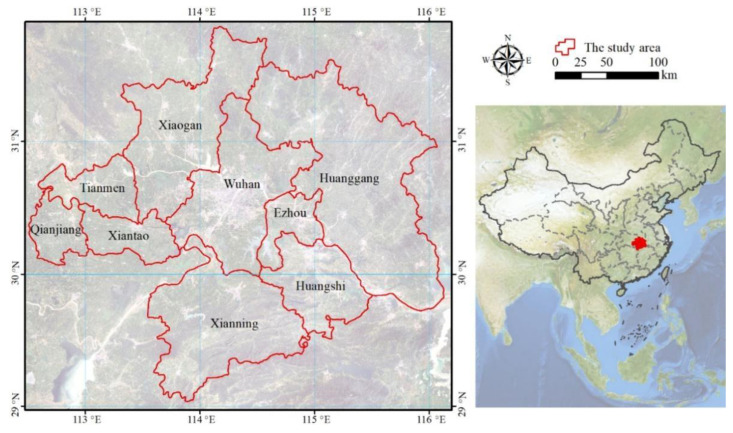
Location of the Wuhan City Circle area in China.

The Wuhan City Circle area is located in central China, and lies between 29°01′–31°87′ and 112°55′–116°23′ north latitude. It is about 57,930 km^2^ (Figure 1). Wuhan City Circle, also called “1 + 8” City Circle, refers to the city clusters formed by Wuhan, the largest city in central China, and eight large and medium-sized cities, including Huangshi, Ezhou, Huanggang, Xiaogan, Xianning, Xiantao, Tianmen and Qianjiang. This region has various topographical landscape types that consist of mountains, hills and plains [24]. Over the past decades the urbanization process has been obvious, mainly reflected in the large increase in built-up land. At the same time, forest land has also increased. However, cropland sharply declined from 2000 to 2015 [25]. The region has a humid mid-subtropical monsoon climate with an average annual temperature of 15.8–17.5 °C, ranging from an average of 3.7 °C during the coolest month (January) to 28.7 °C during the warmest month (July). The frost-free period is usually 211–272 days. The average annual precipitation is 1269 mm.

### 2.2. Data and Processing

Data used in this study mainly included land use maps, climate data (e.g., precipitation, temperature, and radiation), MODIS-NDVI (Normalized Difference Vegetation Index derived from Moderate-resolution Imaging Spectroradiometer) images, and DEM (Digital Elevation Models) (Table 1). Land use maps from 2000, 2005, 2010, and 2015 were downloaded from the Resource and Environment Science and Data Center (https://www.resdc.cn/, accessed on 1 October 2018), and were derived from Landsat TM/ETM+/OLI (Thematic Mapper/Enhanced Thematic Mapper/Operational Land Imager) images. The land use types include 10 classes: paddy field, dry land, arboreal forest, shrub, open forestland 1, open forestland 2, grassland, water, built-up land, and bare land. The details of each land use type can be seen in Table 1.

The monthly climate data (i.e., precipitation, temperature, and radiation) for 2000–2015 were obtained from the National Tibetan Plateau Third Pole Environment Data Center (TPDC) [26]. Based on the time-series MODIS-NDVI images, we used the maximum value compositing algorithm to obtain the monthly NDVI at pixel level.

### 2.3. The CASA Model

The CASA model is a light use efficiency model driven by remote sensing data, temperature, precipitation, solar radiation, vegetation types and soil types [16]. Model simulations use a monthly time step at the regional scale. The monthly NPP is calculated from absorbed photosynthetic active radiation (APAR) and light use efficiency (ε) [17]. APAR depends on the total solar radiation (SOL) and the fraction of photosynthetically active radiation absorbed by the vegetation canopy (FPAR), and is described by Equation (1):

(1) APAR(x, t)=SOL(x, t)× FPAR(x, t)×0.5

where *t* is time (i.e., month) and *x* is spatial location (i.e., pixel). The constant 0.5 represents the proportion of solar effective radiation available to vegetation in the total solar radiation [18]. In this study, FPAR was estimated by Equation (2), as follows:

(2) $$\mathrm{FPAR}\left(x,t\right)=\frac{\mathrm{FPAR}{\left(x,t\right)}_{\mathrm{NDVI}}+\mathrm{FPAR}{\left(x,t\right)}_{\mathrm{SR}}}{2}$$

where FPAR(*x, t*)_NDVI_ and FPAR(*x, t*)_SR_ are FPAR calculated by NDVI (see Equation (3)) and SR (see Equation (4)) in *x* pixel and *t* month, respectively [19].

(3) $$\mathrm{FPAR}{\left(x,t\right)}_{\mathrm{NDVI}}=\frac{\left(\mathrm{NDVI}\left(x,t\right)-{\mathrm{NDVI}}_{i,\min}\right)\left({\mathrm{FPAR}}_{\max}-{\mathrm{FPAR}}_{\min}\right)}{\left({\mathrm{NDVI}}_{i,\max}-{\mathrm{NDVI}}_{i,\min}\right)}+{\mathrm{FPAR}}_{\min}$$

(4) $$\mathrm{FPAR}{\left(x,t\right)}_{\mathrm{SR}}=\frac{\left(\mathrm{SR}\left(x,t\right)-{\mathrm{SR}}_{i,\min}\right)\left({\mathrm{FPAR}}_{\max}-{\mathrm{FPAR}}_{\min}\right)}{{\mathrm{SR}}_{i,\max}-{\mathrm{SR}}_{i,\min}}+{\mathrm{FPAR}}_{\min}$$

In this study, the maximum and minimum values of FPAR were 0.950 (FPAR_max_) and 0.001 (FPAR_min_) in the Wuhan City Circle area, respectively. NDVI_*i*,min_ and NDVI_*i*,max_ refer to the minimum and maximum values of NDVI for the land use type *i* in month *t* (Table 2). SR_*i*,min_ and SR_*i*,max_ refer to the minimum and maximum values of SR for land use type *i* in month *t* (Table 2). SR(*x*, *t*) is the simple ratio of NDVI in *x* pixel and *t* month, and is calculated by NDVI(*x, t*) following Equation (5) [19]:

(5) $$\mathrm{SR}\left(x,t\right)=\frac{1+\mathrm{NDVI}\left(x,t\right)}{1-\mathrm{NDVI}\left(x,t\right)}\;$$

where NDVI(*x, t*) is NDVI in *x* pixel and *t* month.

The light use efficiency (ε) is the efficiency of vegetation in converting absorbed photosynthetic effective radiation into organic carbon [27]. It is mainly affected by temperature and moisture, as in Equation (6):

(6) $$\varepsilon \left(x,t\right)=T_{\varepsilon 1}\left(x,t\right)\times T_{\varepsilon 2}\left(x,t\right)\times W_{\varepsilon }\left(x,t\right)\times \varepsilon_{\max}$$

where T_ɛ1_(*x, t*) and T_ɛ2_(*x, t*) are temperature stress coefficients, W_ɛ_(*x, t*) is the moisture stress coefficient, and ε_max_ is the maximum light use efficiency as determined by the empirical method [28] (Table 2). More detailed information on the CASA model is available in [20,27].

### 2.4. Scenario Design

In this study, we estimated the total carbon sequestration (CS) using Equation (7):

(7) CS=NPP × Area

We designed six scenarios to decouple and analyze the effects of land use change and climate change on carbon dynamics in the Wuhan City Circle area from 2000 to 2015 [20]. The overall effect on carbon dynamics (i.e., ΔAll) includes the effect of climate change (i.e., ΔClimate) and the effect of land use change (i.e., ΔLUCC) in this study (see Equation (8)):

(8) ΔAll=ΔClimate+ΔLUCC

Based on scenario A, we removed the effect of climate change and estimated the impact of ΔLUCC on carbon dynamics during 2000–2015, while ΔClimate could also be calculated by the effect of overall and land use changes (Table 3).

For climate change, we designed three different scenarios: no change in precipitation (Scenario B), no change in temperature (Scenario C), and no change in radiation (Scenario D). Based on these scenarios, we estimated the respective effects of precipitation, temperature, and radiation on carbon dynamics. These were recorded as ΔPrecipitation, ΔTemperature, and ΔRadiation (Table 3).

For land use change, we estimated ΔLUCC following Equation (9):

(9) ΔLUCC=ΔAfforestation+ΔUrbanization+ΔOthers

where ΔAfforestation is the effect of afforestation on carbon sequestration as estimated based on scenario E (Table 3) and ΔUrbanization is the effect of urbanization on carbon sequestration as estimated based on scenario F (Table 3). Based on the above scenarios, we were also able to calculate ΔOthers.

## 3. Results

### 3.1. Spatial and Temporal Variations of Carbon Dynamics

**Figure 2:**
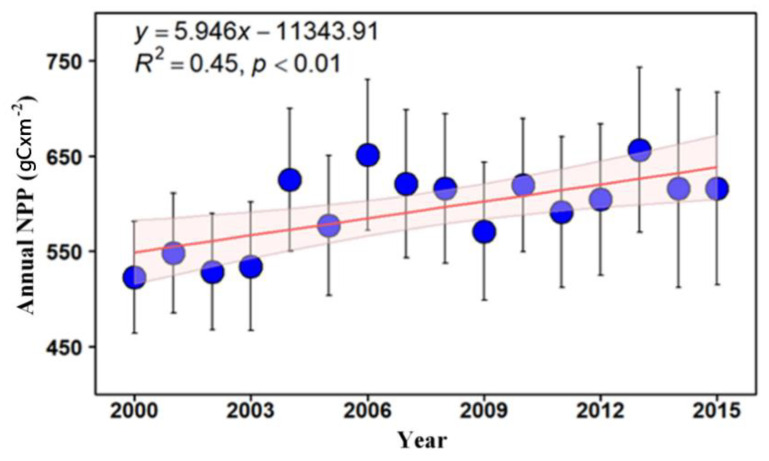
Temporal variation of annual NPP (gC × m^−2^) for the whole Wuhan City Circle area from 2000 to 2015. Note: The red line indicates the linear fitting for the period of 2000 to 2015. The *p* value documents the significance. Error bars extending from the means document the standard deviation of Annual NPP, while the light red region represents the 95% confidence intervals of the linear model.

Our simulation results show that the annual NPP increased from 522.63 gC × m^−2^ to 615.82 gC × m^−2^ between 2000 and 2015 in the Wuhan City Circle area (Figure 2). The maximum NPP occurred in 2013, in which the annual NPP was 655.25 gC × m^−2^. This was followed by 2016, with an annual NPP of 652.56 gC × m^−2^. In contrast, the NPP in 2000, 2002 and 2013 was relatively low, at 522.63 gC × m^−2^, 528.64 gC × m^−2^ and 535.12 gC × m^−2^, respectively. According to the trend analysis of the NPP time series, the annual NPP demonstrated an increasing trend with a growth rate of 5.94 gC × m^−2^ × yr^−1^ in the Wuhan City Circle area.

Although annual NPP increased in all cities of Wuhan City Circle area from 2000 to 2015, there were significant differences in the growth rate between different cities. Comparing 2000 with 2015, Huanggang city had the highest increase in NPP (i.e., 25.27%) in the study area, followed by Xianning and Tianmen, which increased by 120.16 gC × m^−2^ and 102.22 gC × m^−2^, respectively. The annual NPP in Xiantao city only increased by 1.78% in the same period (Table 4). In addition, the NPPs of Ezhou, Qianjiang, Wuhan, Xianning and Xiantao were the largest in 2006, while those of Huanggang, Huangshi, Tianmen and Xiaogan were the highest in 2013. Similarly, the NPP was lowest in 2003 in several cities, excepting Xianning, Huanggang and Huangshi, where it was lowest in 2000. Comparing the annual NPP of the nine cities in the Wuhan City Circle from 2000 to 2015, the maximum NPP was 737.28 gC × m^−2^, which occurred in 2013 in Tianmen city, and the smallest NPP was 399.02 gC × m^−2^, which occurred in 2003 in Ezhou city.

**Figure 3:**
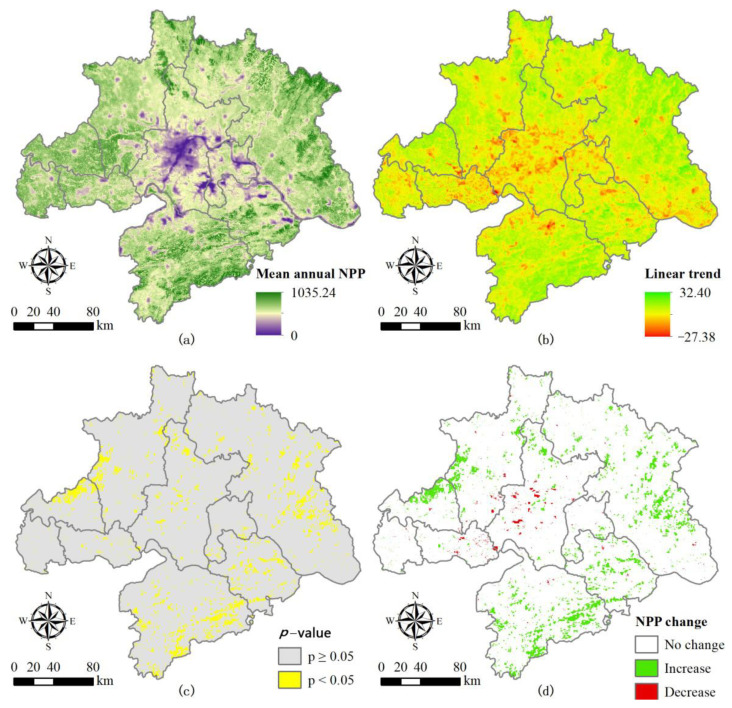
Spatiotemporal variations of annual NPP (gC × m^−2^) in the Wuhan City Circle area at the pixel level. Average annual NPP (**a**), change trends in annual NPP (**b**), the *p*−value of *t*−test for the modeled slopes (**c**), and the NPP change types (**d**). Note: The least-square linear regression model was applied to analyze the temporal variation of annual NPP (Net Primary Productivity) from 2000 to 2015 for each pixel, and the changing trend is described by the modeled slope, i.e., *a* in Figure (**b**). In Figure (**d**), No change documents that annual NPP did not change (*a* = 0 and *p* ≤ 0.05), or that the change was not significant (*a* ≠ 0 and *p* > 0.05).

To explain and analyze the spatial variation of annual NPP in the Wuhan City Circle area, we analyzed the average and linear trend of NPP from 2000–2015 pixel by pixel (Figure 3). We found that the annual NPP was lower in the central region than in the others (Figure 3a), and that the maximum decrease rate was −27.38 gC × m^−2^ × yr^−1^ in the middle of the study area (Figure 3b). Conversely, the annual average NPP was higher in regions outside the central region, especially in Huanggang and Xianning, which had the highest annual average NPP at 1035.24 gC × m^−2^. The maximum growth rate in these areas was up to 32.40 gC × m^−2^ × yr^−1^. Consequently, the annual NPP showed distinct spatial heterogeneity in the Wuhan City Circle area. As a result, the *p*-value of the *t*-test for the modeled slope was less than 0.05 in these study areas (Figure 3c). Only 7.79% of the total area showed an insignificant change in annual NPP during 2000–2015, while more than 90% of the total area showed no change in annual NPP at the 0.05 statistically significant level. Meanwhile, the changes in the annual NPP were significant in the northwest and southeast of the Wuhan City Circle area. Moreover, we defined three NPP change types based on the linear trend of annual NPP and its significance. The reduced areas were mainly distributed in Wuhan and Xiantao. Meanwhile, the increased areas were mainly distributed in Huanggang, Huangshi, Xianning, and at the junction of Xiaogan and Tianmen (Figure 3d). Consequently, 7.24% of the total area (4196.06 km^2^) presented a significant increase in annual NPP at the 0.05 statistically significant level, and only 0.55% of the total area (320.95 km^2^) showed a significant decrease in annual NPP during 2000–2015.

### 3.2. Effects of Climate Change on Carbon Dynamics

Based on our scenario simulations, we found that the effect of climate change on carbon sequestration was −73.3 × 10^10^ gC in the whole Wuhan City Circle area from 2000 to 2015 (Table 5). Our simulation results indicated that Ezhou, Huanggang and Wuhan showed positive effects from climate change on carbon sequestration of 9.02 × 10^10^ gC, 20.2 × 10^10^ gC and 12.2 × 10^10^ gC, respectively, during 2000–2015. In contrast, other cities showed negative effects from climate change on carbon sequestration. The lowest impact of climate change on carbon sequestration was found in Xianning, with −51.9 × 10^10^ gC, followed by Xiaogan with −24.6 × 10^10^ gC.

The annual average temperature of the nine cities revealed that only the temperature in Huangshi decreased, at a rate of −0.005 per year during 2000–2015, while it increased at various rates in all other regions in the Wuhan City Circle (Table S1). Overall, the annual average temperature in Wuhan City Circle was the highest in 2007 and 2013, at 19.24 °C and 19.16 °C, respectively, while the lowest was 17.81 °C in 2012. Similarly, only Qianjiang, Tianmen and Xiaogan had declining annual precipitation, at a rate of −1.67 mm, −2.47 mm and −5.99 mm, respectively (Table S2). In contrast, the annual precipitation in Ezhou has the greatest variation, increasing at a rate of 35.88 mm per year. The annual precipitation in Wuhan City Circle was the highest in 2002, at 2326.21 mm, and the lowest in 2007, at 1468.26 mm. In terms of annual radiation, Qianjiang, Tianmen and Xiantao saw a decrease at a rate of −3.34 MJ × m^−2^, −1.30 MJ × m^−2^ and −0.34 MJ × m^−2^ per year, respectively, while other regions saw an increase at various rates (Table S3). The annual radiation of Wuhan City Circle was the highest in 2006 at 5793.76 MJ × m^−2^ per year, and the lowest in 2003 at 4068.50 MJ × m^−2^ per year.

The changes in precipitation and temperature had a negative effect, by −71.2 × 10^10^ gC and −78.2 × 10^10^ gC, respectively, on carbon sequestration in the whole study area from 2000–2015 (Table 5). Specifically, changes in precipitation and temperature had positive effects on carbon sequestration in Huanggang and Wuhan, while they had negative effects in the other cities. We also found that Xianning showed the lowest effect from precipitation (−31.0 × 10^10^ gC) and temperature (−52.7 × 10^10^ gC). However, radiation in the Wuhan City Circle area had a positive effect of 76.1 × 10^10^ gC on total carbon sequestration in the same period. Contrary to the trends in precipitation and temperature, radiation changes in Huanggang and Wuhan had a negative impact on carbon sequestration. The highest effect of radiation (31.9 × 10^10^ gC) on carbon sequestration was found in Xianning (Table 5). In addition, the highest simulated value of the effect of temperature was 20.3 × 10^10^ gC in Huanggang, while the lowest effect of radiation on carbon sequestration was −11.3 × 10^10^ gC, in Wuhan (Table 5).

### 3.3. Effects of Land Use Change on Carbon Dynamics

According to our scenarios, the results indicate that the effect of land use change on carbon sequestration was 480 × 10^10^ gC in the whole Wuhan City Circle area during 2000–2015 (Table 6). Overall, in this period, the largest increase in land use type was in built-up land, which was 1496.36 km^2^. This is followed by water at 630.76 km^2^ (Table S4). Moreover, the most obvious decreases of land use types in the Wuhan City Circle area from 2000 to 2015 were dry land (i.e., −1000.92 km^2^) and paddy field (i.e., −948.68 km^2^) (Table S4). However, afforestation and urbanization had little effect on carbon sequestration in the Wuhan City Circle from 2000–2015, at 6.05 × 10^10^ gC and −6.29 × 10^10^ gC, respectively (Table 6). The most affected by afforestation was Xiaogan, with 1.38 × 10^10^ gC, while the areas with the highest effect of urbanization were Xianning and Huanggang with −2.64 × 10^10^ gC and −2.04 × 10^10^ gC, respectively. In contrast, ΔOthers played a decisive role in the influence of carbon sequestration, with 479.0 × 10^10^ gC (Table 6). The city most affected by ΔOthers was Huanggang, with a value of ΔOthers of 164.0 × 10^10^ gC; the least affected was Ezhou, with 7.20 × 10^10^ gC.

## 4. Discussion

### 4.1. Relationship between Carbon Dynamics and Climate Change

Climate change is an important driving factor affecting regional NPP changes [29]. In this study, climate change showed a negative effect on carbon sequestration in the whole Wuhan City Circle area between 2000 and 2015. We found that this negative effect mainly came from an increase in temperature and precipitation. Similar results were found by Khalifa et al. [30], who indicated that temporal variations in NPP mainly depend on changes in climatic factors such as temperature, precipitation, and radiation. According to the change trend analysis, the annual average temperature in the Wuhan City Circle area showed an insignificant increasing trend of 0.005 °C × year^−1^ (Table S1). Some previous studies have reported that carbon sequestration in forest ecosystems can decrease with increasing temperatures if the region has good moisture [31,32]. This can be attributed to carbon loss due to soil respiration, as the increase in temperature usually directly enhances both autotrophic and heterotrophic soil respiration [33]. As an important factor in climate, precipitation is also a key driving factor of forest NPP [34,35]. The average precipitation in the Wuhan City Circle area was 1872.50 mm × year^−1^ between 2000 and 2015, and the annual average precipitation also showed an increasing trend of about 10 mm × year^−1^ (Table S2). This can intensify soil erosion and thus soil organic carbon denudation, transport and deposition [36]. In addition, soil erosion can lead to soil nutrient loss, thus indirectly affecting ecosystem productivity and carbon sink function [36].

**Figure 4:**
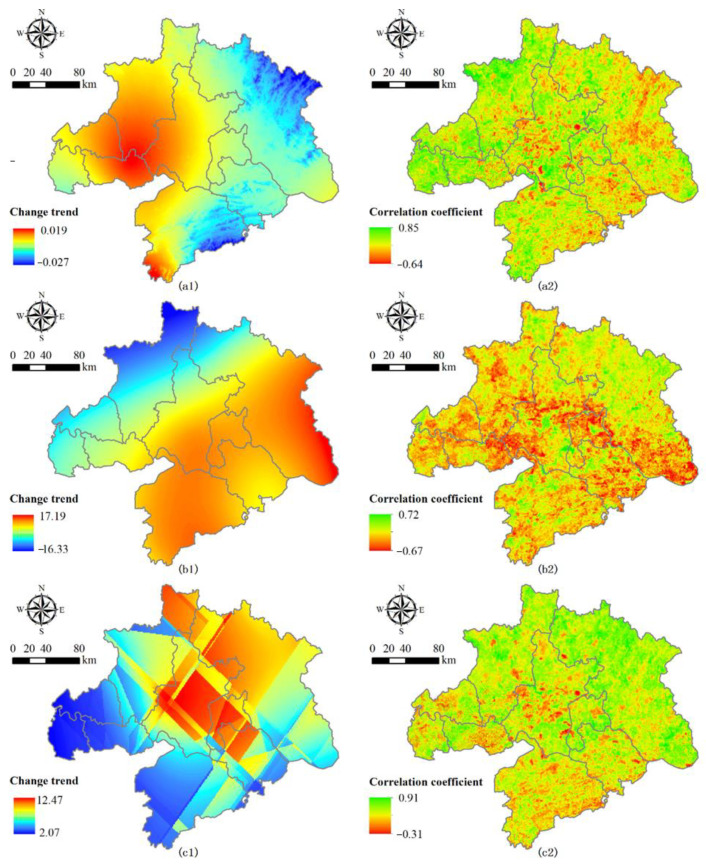
Climate change trends and Spearman’s rank correlations between the annual NPP and climate variables for the period 2000−2015. (**a**) Temperature, (**b**) Precipitation, (**c**) Radiation.

The regions with significant temperature increases were mainly located in the western part of the study area, which showed the highest change rate at 0.019 °C (Figure 4(a1)), while the temperature decreased significantly in Huanggang and Xianning. The Spearman’s rank correlation between NPP and annual mean temperature was significant. The spatial explicit correlation coefficients were positive in the northwest regions of Wuhan City Circle, and the maximum value of the correlation coefficient was 0.85 in this area (Figure 4(a2)). However, the spatial explicit correlation coefficients were negative in the central and eastern regions of Wuhan City Circle (Figure 4(a2)), while the minimum value of the correlation coefficient between the annual NPP and temperature was −0.64 in these areas. At the same time, precipitation caused NPP to decrease in most regions, excepting only the northeastern regions (Figure 4(b2)). The minimum value of the correlation coefficient between the annual NPP and precipitation was −0.67 from 2000 to 2015. However, the precipitation increased in most areas of Wuhan City Circle (except for the northwest), and the maximum growth in precipitation was up to 17.19 mm (Figure 4(b1)).

Radiation is known to have very important effects on many biological processes, such as photosynthesis and growth in terrestrial vegetation [3]. In the Wuhan City Circle area, the average annual radiation was 4713.27 MJ × m^−2^ between 2000 and 2015, and the change rate was 1.72 MJ × m^−2^ × year^−1^ (Table S3). Usually, forest carbon sequestration was positively related to radiation [5,20,37]. For example, Jiang et al. [38] showed that reduction in cloud cover increased solar radiation reaching the Earth’s surface, thereby increasing NPP. Mercado et al. [39] demonstrated that solar radiation was the main driver of plant photosynthesis. Thus, the increase of solar radiation is beneficial to the formation of carbon sinks. This is completely consistent with our simulation results, as NPP increased with radiation in the northeastern regions but decreased in the more central regions (Figure 4(c2)). However, there was a significant increasing trend in radiation in both the central and northeastern regions of the study area, with a maximum increment of 12.47 MJ × m^−2^ (Figure 4(c1)). The reason for this difference is mainly due to the central area of Wuhan City Circle being a key part of urban expansion, with less vegetation coverage. The maximum value of the correlation coefficient between annual NPP and radiation was 0.91. This value is higher than both temperature and precipitation in the Wuhan City Circle area during the same period.

### 4.2. Land Use Change Altered Carbon Sequestrations

**Figure 5:**
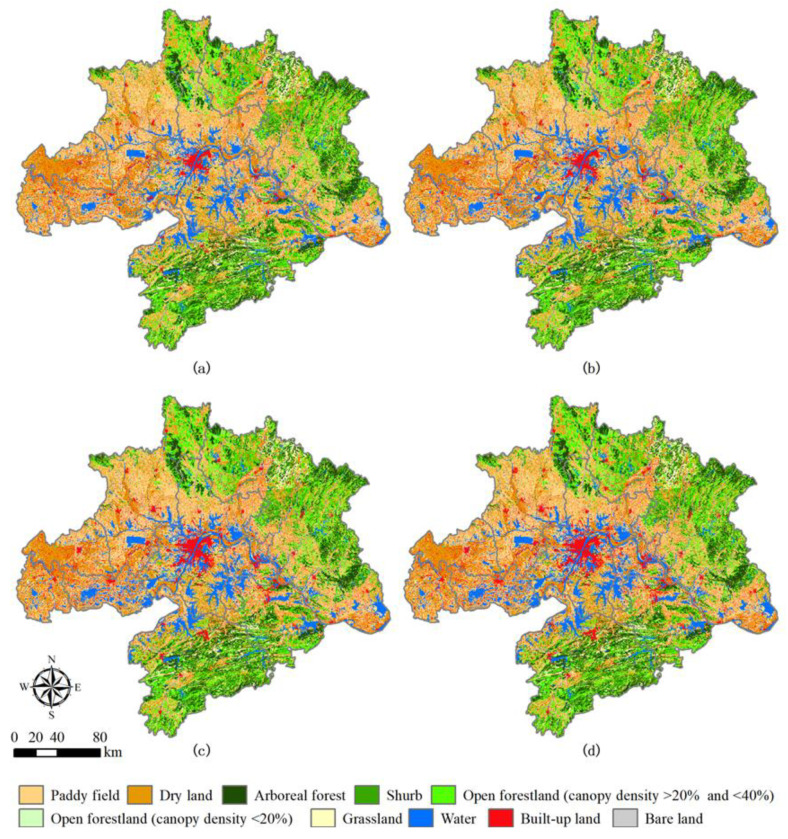
Spatial explicit land use maps of the Wuhan City Circle area in 2000 (**a**), 2005 (**b**), 2010 (**c**) and 2015 (**d**).

Forest plays a very important and unique critical role in terrestrial carbon sequestration and slowing down global warming [40,41,42]. In this study, forestland had the highest annual NPP from the spatial distribution map of land use (Figure 5) and the spatial distribution map of annual NPP (Figure 3a). The Afforestation area was 395.39 km^2^ from 2000 to 2015, while the effect of afforestation had a positive value of 6.05 × 10^10^ gC. Moreover, ΔOthers had the highest effect on carbon sequestration, 479.0 × 10^10^ gC in the study area. The major reason for this was that a large amount of forestland had been protected and grown naturally during these years. We know that the factors affecting forest carbon sequestration mainly include climate condition, forest composition (i.e., tree age, tree species, and density) and forest management [43,44,45]. However, limited by the acquisition of forestry survey data, we were unable to analyze the impact of specific forest management level and stand structure on regional carbon sequestration in the Wuhan City Circle area. Because the increase of temperature and precipitation brings negative effects, we think that the positive effects of afforestation may come with increasing forest age and effective radiation.

In addition, the positive effects of afforestation on carbon sequestration in the Wuhan City Circle area during 2000–2015 were almost equal to the negative effects of urbanization (i.e., −6.29 × 10^10^ gC). However, built-up land increased by 1496.36 km^2^, which was almost 20 times the reduced area of forestland (Table S4). Actually, we also found that the built-up land increased by urbanization mainly came from the reduction of cropland (i.e., −1949.6 km^2^, which includes paddy field and dry land) in the Wuhan City Circle area (Figure 5). Specifically, 1244.43 km^2^, 222.8 km^2^ and 127.2 km^2^ of cropland, forest land and water were transferred to built-up land during 2000–2015 (Table S5), resulting in a significant loss of NPP. These results are similar to those reported by Liu et al. [45], who also concluded that the increase in land use types with higher photosynthetic productivity offset a certain amount of NPP loss despite the rapid encroachment of built-up land on cropland. Therefore, 310.75 km^2^ and 51.66 km^2^ of crop land and grassland were transformed into forest land under the influence of the reforestation policy (Table S5), which effectively compensated for the negative impact of urban expansion on NPP in the Wuhan City Circle area.

Other land use changes were mainly reflected in the substantial decrease in crop land and increase in water area, (i.e., 630.76 km^2^) in the Wuhan City Circle area between 2000 and 2015 (Table S4). Of this, 887.67 km^2^ of cropland and 103.44 km^2^ of bare land were converted to water during the study period (Table S5). Between 2000 and 2015, 260.69 km^2^ of water and 164.36 km^2^ of forest land were also converted to crop land. However, the transfer of crop land in was much smaller than the transfer out, so it mainly showed a decreasing trend. We think that increased precipitation, complex human activities and the rugged topography are the main reasons for these changes.

### 4.3. Land Use Suggestions and Landscape Planning

**Figure 6:**
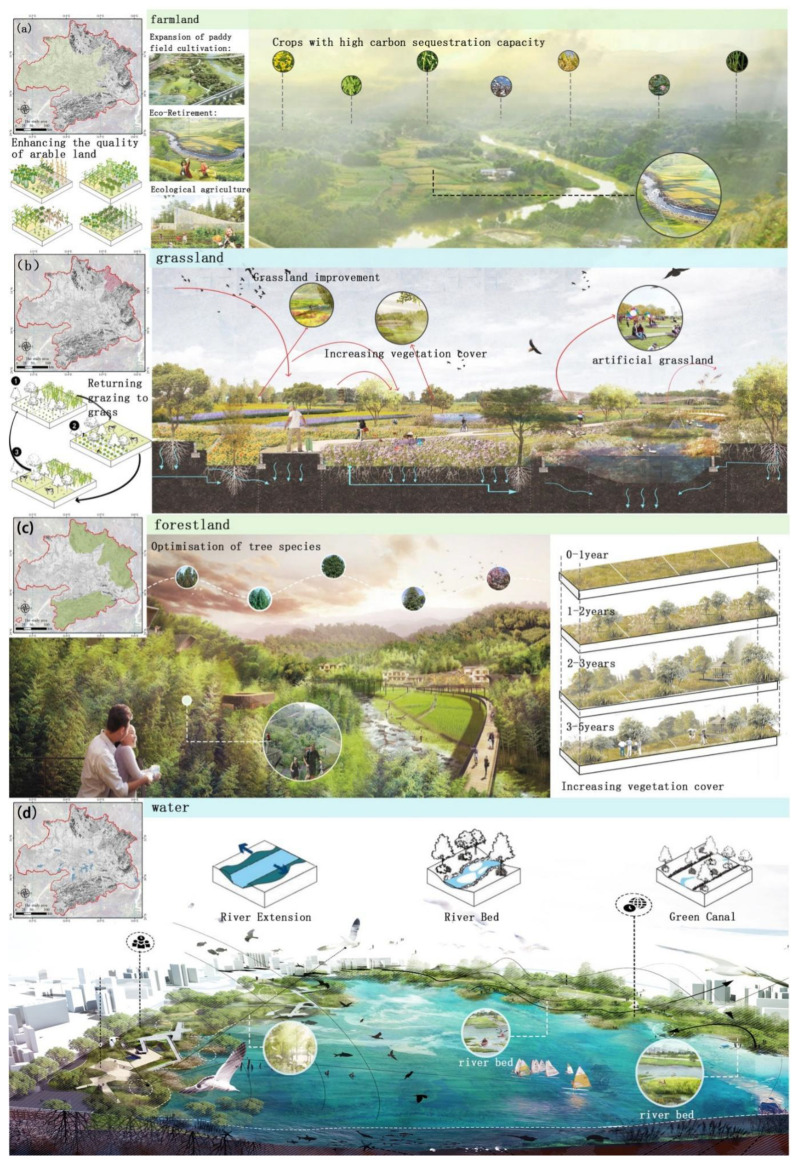
Landscape planning for farmland (**a**), grassland (**b**), forestland (**c**) and water (**d**) in the Wuhan City Circle area.

There are four typical landscapes, i.e., forest, water, farmland and grassland, in the Wuhan City Circle area. We proposed different targeted and creative design proposals to optimize the scale structure and spatial layout for these landscapes by integrating hill, water, forest, field, lake and grass management (Figure 6). These landscape measures could reduce the occupation and degradation of ecological land and increase ecosystem protection and restoration, which could enhance the carbon sequestration capacity and efficiency of regional ecosystems.

Although some forests have converted into cropland, the amount of crop land transferred in is much smaller than the amount transferred out. Thus, the red line of crop land protection should be strictly adhered to and the quality of farmland and ecological construction should be strengthened. Landscape planning tries to reduce the occupation of paddy fields and, under the premise of soil and water balance, moderately expand the area of cropland under cultivation in order to increase the carbon sink of arable soils (Figure 6a).In addition, comprehensive treatments such as ecological fallowing, soil restoration, treating surface source pollution, and reducing groundwater extraction should be implemented in the regions where environmental problems such as agricultural pollution, water scarcity and serious soil erosion exist, so as to improve the quality of arable land and the function of soil carbon sequestration. At the same time, we suggest implementing combined food storage with respect to the land and use of nutrients, drawing on the experience of typical countries and regions in the spatial and temporal allocation of fallow land, while also taking into account the matching of water and soil pollution and ecological vulnerability in order to reasonably determine the spatial and temporal allocation of fallow crop rotation.

For the grassland ecosystem, limiting overgrazing and optimizing grass species structure can have an important positive impact on maintaining and increasing carbon storage in terrestrial ecosystems (Figure 6b). It is recommended that zoned rotational grazing, grazing bans and seasonal grazing rests should be implemented in order to achieve grazing reversion and grass–livestock balance. Grassland improvement is implemented through moderate fertilization, irrigation, and improved species selection, and the construction of artificial grasslands can be strengthened to improve the comprehensive vegetation cover of grasslands and increase the organic matter content of soils.

The Wuhan City Circle area includes a large forested area, and forests play a key and unique role in carbon sequestration and mitigating global warming. According to our results, forests have the highest annual NPP, and increasing forest cover could significantly promote carbon sequestration (Figure 6c). Meanwhile, forest management measures such as optimized tree species structure, rotation period selection, and fertilizer application programs are recommended, as these are very important for increasing the amount of vegetation and soil carbon sequestration in forest ecosystems. Some studies have reported that the soil organic carbon stock of broad-leaved forests is significantly higher than that of coniferous forests, and that mixed configurations should be planted according to the shade tolerance and successional order of tree species. The rotation periods should be extended appropriately, and reasonable rotation periods and volumes should be determined according to tree species in each region in order to make full use of forest carbon sequestration potential. In terms of forest management, artificial afforestation for forest regeneration has become a way to increase soil carbon sequestration under human control. Management measures such as fertilization, irrigation, forest thinning, forest weed management and controlled fires have been adopted to strengthen forestry management and change the determinants of afforestation area, the rotation period of tree species, and average annual growth in order to enhance the level of afforestation and sink in the region.

For the waterscape, we suggest building green ecological corridors, ecological dams and ecological floating islands to improve carbon sequestration capacity (Figure 6d). These measures could enhance ecological habitats for plants and aquatic plants with strong carbon sequestration capacity, so as to enhance the carbon sequestration capacity of the watershed.

### 4.4. Uncertainties and Limitations

In this study, some uncertainties in our simulation results must also be considered. First, uncertainty in the input of climate data (i.e., temperature, precipitation, and radiation) comes from the ANU–Spline statistical interpolation based on meteorological observation, reanalysis data and satellite remote sensing data [26]. Second, we did not consider the effects of atmospheric processes. For example, both the acceleration of nitrogen deposition and the increase of atmospheric carbon dioxide concentration may affect simulated NPP at the regional scale [46,47,48,49]. Third, the simulated NPP comes from the downscaling of MODIS-NDVI products [50]. There is no doubt that more accurate data can improve the performance of the CASA model. However, this study focuses on the effects of climate change and land use change on regional carbon sequestration through relative comparison. Therefore, the influence of the above shortcomings on the accuracy of our simulated results can be largely ignored.

## 5. Conclusions

Our simulations showed that the annual NPP of the Wuhan City Circle area followed an increasing trend from 2000 to 2015, while the total carbon sequestration was 3027.6 × 10^10^ gC in 2000 and 3567.45 × 10^10^ gC in 2015. Based on our scenario simulations, we found that land use changes contributed to total carbon sequestration by 480 × 10^10^ gC in the whole study area during the period of 2000–2015. However, climate change had a negative effect on total carbon sequestration of −73.3 × 10^10^ gC in the same area and period. Both precipitation and temperature released carbon by −71.2 × 10^10^ gC and −78.2 × 10^10^ gC, respectively; however, radiation increased carbon by 76.1 × 10^10^ gC. In terms of spatial distribution, we found that the negative effect was mainly in the central region, while the positive effects were mostly in the surrounding region. Moreover, the effects of climate change on carbon sequestration in the Wuhan City Circle area were much less than the effect of land use change between 2000 and 2015. The effects of afforestation and urbanization on carbon sequestration offset each other in the study area, which indicates that the government was simultaneously implementing afforestation and urbanization during 2000–2015. However, the negative effects of the large reduction in paddy fields and dry land on carbon sequestration needs to be given great attention.

## Figures and Tables

**Table 1:** The information of land use type.

Class I	Class II	Description
Crop land	Paddy field	Cultivated land with water sources and irrigation facilities, which can be irrigated normally in typical years to plant aquatic crops such as rice and lotus root; also includes cultivated land with rice and dry land crop rotation.
	Dry land	Cultivated land that grows crops by natural precipitation; dry cultivated land with water sources and irrigation facilities that can be irrigated normally in typical years; cultivated land mainly for growing vegetables; normal rotation of fallow land and rotation rest land.
Forest land	Arboreal forest	Natural and plantation forest with canopy density >40%; includes timber forest, economic forest, shelter forest and other woodlands.
	Shrub	Low and shrub woodland with canopy density >40% and height below 2 m.
	Open forest land 1	Forest with canopy density 20–40%.
	Open forest land 2	Forest with canopy density <20%.
Grassland	-	Natural, improved and mowed grasslands with dense growth;
Water	-	natural waters and water conservation facilities.
Built-up land	-	Urban and rural residential land; mining land and other transportation land outside urban and rural areas.
Bare land	-	Land with surface soil coverage and vegetation coverage <5%.

**Table 2:** Parameters of the CASA model for different land use types in the Wuhan City Circle area.

Land Use Type	NDVImax	NDVImin	SRmax	SRmin	εmax
Paddy field	0.7994	0.0765	14.393	1.166	0.729
Dry land	0.8824	0.0765	17.002	1.166	0.821
Arboreal forest	0.8979	0.0765	18.793	1.166	0.985
Shrub	0.8983	0.0765	18.666	1.166	0.756
Open forestland 1	0.8889	0.0765	18.589	1.166	0.779
Open forestland 2	0.7994	0.0765	14.393	1.166	0.679
Grassland	0.6653	0.0765	12.576	1.166	0.429
Water	0.5044	0.0765	8.97	1.166	0.429
Built-up land	0.5044	0.0765	8.97	1.166	0.429
Bare land	0.5044	0.0765	8.97	1.166	0.429

Note: NDVImin and NDVImax refer to the minimum and maximum values of NDVI for each land use type. SRmin and SRmax refer to the minimum and maximum values of SR for each land use type. The εmax refers to the maximum value of light use efficiency.

**Table 3:** Scenario design for quantifying the effects of climate change and land use change on carbon dynamics in the Wuhan City Circle area from 2000 to 2015.

Scenario	Explanation/Purpose
A	Keeping the climate conditions (precipitation, temperature, and radiation) at the same level as in 2000, the potential carbon sequestration in 2015 (CSA) was calculated using the land use map and NDVI images from 2015. The effect of all land use/cover changes was calculated as ΔLUCC = CSA − CS2000. On the one hand, we knew that the overall effect equals the difference between the actual CS in 2000 and 2015, i.e., Δ = CS2015 − CS2000. On the other hand, we hypothesized that the overall effect only consisted of the effects of climate change and land use changes, i.e., Δ = ΔClimate + ΔLUCC. Therefore, we also calculated the effect of climate change using the equation ΔClimate = CS2015 − CSA.
B	Keeping the precipitation at the same level as in 2000, the potential CS in 2000 (CSB) was estimated by using the land use map and NDVI images from 2015. We then calculated the effect of precipitation according to the equation ΔPrecipitation = CS2015 − CSB.
C	Keeping the temperature at the same level as in 2000, the potential CS in 2015 (CSC) was estimated by using the land use map and NDVI images from 2015. We then calculated the effect of temperature according to the equation ΔTemperature = CS2015 − CSC.
D	Keeping the radiation at the same level as in 2000, the potential CS in 2015 (CSD) was estimated by using the land use map and NDVI images from 2015. We then calculated the effect of radiation according to the equation ΔRadiation = CS2015 − CSD.
E	Keeping the climate conditions and the NDVI values for afforestation pixels at the same level as in 2000, we calculated the potential CS in 2015 caused by land use changes, except for afforestation (CSE). We then calculated the effect of afforestation according to the equation ΔAfforestation = CSA − CSE.
F	Keeping the climate conditions and the NDVI values for urbanization pixels the same as the level in 2000, we calculated the potential CS in 2015 caused by land use changes, except for urbanization (CSF). We then calculated the effect of urbanization according to the equation ΔUrbanization = CSA − CSF.

**Table 4:** Temporal variations of annual NPP (gC × m^−2^) for nine cities in Wuhan City Circle from 2000 to 2015.

Year	Ezhou	Huanggang	Huangshi	Qianjiang	Tianmen	Wuhan	Xianning	Xiantao	Xiaogan
2000	412.82	533.29	481.37	596.26	597.56	441.18	531.69	568.28	563.21
2001	447.21	575.40	526.53	602.73	598.01	446.40	566.70	564.88	572.87
2002	410.72	548.25	503.81	601.13	596.05	432.62	547.29	562.07	553.36
2003	399.02	564.40	542.19	538.07	571.96	417.63	585.89	533.39	543.78
2004	523.80	672.87	603.71	641.69	669.17	503.49	638.52	630.06	648.63
2005	470.33	621.09	531.77	647.30	656.35	473.77	569.00	607.78	596.03
2006	528.62	679.09	611.07	727.34	735.14	529.47	679.10	677.11	679.78
2007	507.29	652.44	591.75	676.03	682.65	493.87	670.15	629.17	632.62
2008	479.46	657.24	606.67	646.33	660.78	489.34	646.11	620.52	637.29
2009	461.16	604.68	578.31	576.58	596.67	442.00	620.19	556.56	589.39
2010	505.42	635.98	623.56	637.93	682.79	494.46	659.56	599.60	665.57
2011	475.27	592.30	613.61	645.82	677.41	458.98	663.37	610.14	603.63
2012	478.37	670.99	573.03	604.10	658.06	474.44	621.01	565.48	620.55
2013	501.86	706.65	625.04	691.18	737.28	508.86	675.53	647.66	698.63
2014	440.79	678.92	572.63	619.46	693.14	459.57	667.87	569.05	636.27
2015	433.54	668.06	575.46	664.90	699.78	463.12	651.85	578.37	656.51
Change rate	2.28	7.88	5.98	3.43	7.13	2.02	7.75	1.30	6.29

**Table 5:** The effects of climate change, precipitation, temperature and radiation on total carbon sequestration (10^10^ gC) for the Wuhan City Circle area between 2000 and 2015. ΔClimate, the effect of climate changes; ΔPrecipitation, the effect of precipitation changes; ΔTemperature, the effect of temperature changes; ΔRadiation, the effect of radiation changes.

City	ΔClimate	ΔPrecipitation	ΔTemperature	ΔRadiation
Ezhou	9.02	−0.04	−0.07	9.13
Huanggang	20.2	5.34	20.3	−5.46
Huangshi	−6.59	−1.53	−6.90	1.84
Qianjiang	−14.2	−11.5	−13.6	10.9
Tianmen	−12.3	−10.0	−11.3	9.04
Wuhan	12.2	10.4	13.1	−11.3
Xianning	−51.9	−31.0	−52.7	31.9
Xiantao	−5.21	−2.38	−4.35	1.52
Xiaogan	−24.6	−30.4	−22.8	28.5

**Table 6:** The effects of land use changes on total carbon sequestration (10^10^ gC) for the Wuhan City Circle area during the period between 2000 and 2015. ΔLUCC, the effect of all land use changes; ΔAfforestation, the effect of non-forest land changes on forest land; ΔUrbanization, the effect of non-construction land changes on construction land; ΔOthers, the effect of other land use changes.

City	ΔLULC	ΔAfforestation	ΔUrbanization	ΔOthers
Ezhou	6.96	0.09	−0.33	7.20
Huanggang	163.0	0.18	−2.04	164.0
Huangshi	20.6	0.16	−1.15	21.6
Qianjiang	27.7	0.51	−0.04	27.2
Tianmen	35.0	0.96	0.21	33.8
Wuhan	35.4	0.97	−0.8	35.2
Xianning	86.0	0.94	−2.64	87.7
Xiantao	13.3	0.86	0.28	12.2
Xiaogan	92.4	1.38	0.22	90.8
Wuhan City Circle	480.0	6.05	−6.29	479.0

## Supplementary Materials

The following are available online at https://www.mdpi.com/article/10.3390/ijerph182111617/s1, Table S1: Temporal variations of annual average temperature (°C) for the nine cities in Wuhan City Circle from 2000 to 2015. Table S2: Temporal variations of annual precipitation (mm) for the nine cities in Wuhan City Circle from 2000 to 2015. Table S3: Temporal variations of annual radiation (MJ × m^−2^) for the nine cities in Wuhan City Circle from 2000 to 2015. Table S4: Area (km^2^) of various land types in Wuhan City Circle in 2000, 2005, 2010, 2015, and changes between 2000 and 2015. Table S5: Land use transfers between 2000 and 2015 in Wuhan City Circle.
