# Supplementary material for: Simulation and Analysis of the Effects of Land Use and Climate Change on Carbon Dynamics in the Wuhan City Circle Area
Source: Int J Environ Res Public Health. 2021 Nov 4;18(21):11617. doi: 10.3390/ijerph182111617 (PMC8582944; doi:10.3390/ijerph182111617)
Supplement: Supplementary file 1 [file ijerph-18-11617-s001.zip › ijerph-1387428-supplementary.pdf]

Table S1. Temporal variations of annual average temperature (°C) for 9 cities and Wuhan City Circle from 2000 to 2015.

	**Ezhou**	**Huanggang**	**Huangshi**	**Qianjiang**	**Tianmen**	**Wuhan**	**Xianning**	**Xiantao**	**Xiaogan**	**Wuhan City Circle**
2000	17.72	18.04	18.20	17.71	17.66	18.06	18.22	17.86	17.68	18.22
2001	18.29	18.24	18.46	18.08	18.04	18.36	18.49	18.19	18.05	18.49
2002	18.24	18.36	18.47	18.05	17.98	18.31	18.46	18.15	17.99	18.47
2003	17.91	18.25	18.33	17.59	17.49	17.99	18.33	17.74	17.50	18.33
2004	18.47	18.45	18.65	18.38	18.27	18.58	18.74	18.44	18.28	18.74
2005	18.15	18.21	18.39	17.91	17.84	18.24	18.40	18.04	17.85	18.40
2006	18.68	18.67	18.89	18.53	18.42	18.79	18.99	18.62	18.43	18.99
2007	18.98	19.07	19.24	18.76	18.69	19.07	19.23	18.89	18.70	19.24
2008	18.21	18.33	18.50	18.12	17.96	18.33	18.56	18.19	17.97	18.56
2009	18.36	18.42	18.62	18.24	18.10	18.48	18.72	18.33	18.12	18.72
2010	18.07	18.17	18.32	17.96	17.86	18.16	18.35	18.02	17.87	18.35
2011	17.89	17.98	18.19	17.61	17.56	18.00	18.23	17.78	17.57	18.23
2012	17.62	17.61	17.73	17.16	17.37	17.70	17.81	17.55	17.43	17.81
2013	18.80	18.80	19.04	18.62	18.49	18.91	19.16	18.71	18.51	19.16
2014	18.48	18.56	18.72	18.00	18.19	18.57	18.69	18.41	18.24	18.72
2015	18.05	18.29	18.03	18.19	18.31	18.32	18.37	18.39	18.32	18.39
Change rate	0.007	0.003	-0.005	0.003	0.014	0.007	0.004	0.013	0.015	0.005

Table S2. Temporal variations of annual precipitation (mm) for 9 cities and Wuhan City Circle from 2000 to 2015.

	**Ezhou**	**Huanggang**	**Huangshi**	**Qianjiang**	**Tianmen**	**Wuhan**	**Xianning**	**Xiantao**	**Xiaogan**	**Wuhan City Circle**
2000	27.77	1191.65	1281.68	1195.89	1183.53	1166.49	1360.46	1216.76	1194.53	1610.33
2001	1074.90	1033.09	1210.90	948.77	879.31	960.08	1259.80	988.09	800.79	1506.96
2002	1664.51	1589.55	1764.49	1554.49	1405.32	1590.01	1988.70	1617.46	1381.31	2326.21
2003	1498.59	1496.75	1552.57	1206.66	1223.04	1468.44	1579.53	1388.65	1364.83	1747.80
2004	1346.45	1285.21	1352.83	1317.56	1285.63	1332.88	1458.61	1408.51	1200.73	1596.57
2005	1204.80	1324.82	1409.44	918.92	946.66	1135.84	1389.30	1007.72	1122.41	1813.24
2006	1096.21	1023.77	1184.54	1022.41	968.94	1016.39	1284.83	1069.42	915.73	1524.87
2007	1186.91	1210.46	1188.53	1069.77	1079.82	1158.26	1203.38	1072.31	1196.60	1468.26
2008	1270.36	1350.85	1287.58	1225.09	1267.70	1321.22	1414.74	1300.95	1372.41	1596.88
2009	1255.94	1241.68	1284.33	1152.04	1097.23	1167.99	1367.71	1252.56	976.79	1572.64
2010	1771.31	1609.68	1880.97	1298.15	1241.50	1584.92	2083.04	1546.23	1244.90	2297.19
2011	1145.24	1036.88	1199.30	931.32	914.75	1038.10	1284.13	1063.38	818.75	1479.82
2012	1467.85	1359.28	1612.93	1132.64	1015.13	1294.54	1790.67	1265.99	958.10	2216.06
2013	1184.00	1162.83	1162.83	1198.54	1154.28	1192.48	1270.21	1230.39	1055.88	1494.38
2014	1456.78	1452.23	1576.89	1046.46	992.52	1296.41	1649.50	1170.30	1074.71	1938.67
2015	1561.51	1546.98	1643.86	1358.69	1318.69	1490.11	1742.27	1506.67	1281.60	1897.69
Change rate	35.88	7.84	8.52	-1.67	-2.47	6.01	11.03	3.54	-5.99	10.09

Table S3. Temporal variations of annual radiation ($MJ \bullet m^{-2}$) for 9 cities and Wuhan City Circle from 2000 to 2015.

	**Ezhou**	**Huanggang**	**Huangshi**	**Qianjiang**	**Tianmen**	**Wuhan**	**Xianning**	**Xiantao**	**Xiaogan**	**Wuhan City Circle**
2000	4222.35	4328.22	4249.08	4126.03	4172.47	4267.11	4211.65	4174.11	4276.52	4262.18
2001	4503.27	4569.59	4486.05	4278.78	4365.00	4502.40	4403.52	4371.86	4501.98	4484.44
2002	4144.72	4223.27	4119.35	4033.84	4104.73	4163.51	4065.87	4077.98	4208.40	4156.48
2003	4088.22	4167.97	4191.66	3817.61	3854.74	4029.11	4118.07	3907.86	3958.81	4068.50
2004	4567.72	4722.91	4517.43	4249.68	4354.98	4551.34	4343.01	4336.47	4579.32	4540.16
2005	4103.02	4260.64	4044.02	3835.26	3938.33	4093.04	3830.64	3890.81	4119.21	4073.90
2006	5803.01	5813.65	5810.13	5764.05	5767.94	5787.85	5789.60	5775.50	5774.78	5793.76
2007	4529.31	4610.38	4464.17	4240.58	4353.94	4530.95	4283.02	4324.42	4533.91	4480.50
2008	4450.87	4504.45	4460.80	4171.58	4255.86	4420.59	4368.60	4279.37	4388.04	4413.40
2009	4285.86	4346.91	4331.90	4177.29	4210.62	4271.51	4288.79	4222.49	4254.96	4291.18
2010	4316.79	4373.31	4343.24	4118.96	4177.51	4294.33	4287.20	4204.21	4277.69	4303.11
2011	4232.27	4279.76	4200.69	4107.30	4171.08	4240.45	4159.60	4164.97	4250.20	4225.37
2012	4329.70	4167.77	4370.57	4278.04	4341.85	4362.80	4386.28	4371.70	4307.99	4296.71
2013	4464.23	4628.83	4420.04	4042.41	4173.13	4431.80	4216.95	4157.56	4440.17	4417.82
2014	4700.81	4836.84	4702.27	4187.61	4308.55	4636.03	4537.35	4362.89	4585.44	4635.80
2015	4262.43	4382.63	4169.39	4064.88	4144.01	4293.45	4076.37	4118.81	4332.23	4255.80
Change rate	4.34	2.51	2.02	-3.34	-1.30	3.32	0.54	-0.34	2.05	1.72

Table S4. Area (km^2) of various land types in Wuhan City Circle in 2000, 2005, 2010, 2015, and changes between 2000 and 2015.

Land use type	2000	2005	2010	2015	Changes (2000 ~ 2015)
Paddy field	18327.81	18005.48	17631.9	17379.13	-948.68
Dry land	11601.07	11453.53	10764.76	10600.15	-1000.92
Arboreal forest	6532.67	6507.78	6423.85	6398.2	-134.46
Shrub	2824.82	2812.84	2832.55	2822.1	-2.72
Open forestland 1	8045.62	8015.53	8064.56	8015.03	-30.59
Open forestland 2	99.85	154.33	196.51	203.99	104.14
Grass land	1442.2	1423.23	1402.13	1396.19	-46.01
Water	5736.13	6015.54	6360.25	6366.89	630.76
Build-up land	3072.72	3301.68	4071	4569.09	1496.36
Bare land	247.1	240.05	182.5	179.23	-67.87

Table S5. Land use transfers between 2000 and 2015 in Wuhan City Circle.

Area (km^2)		2015					
		**Crop land**	**Forest land**	**Grassland**	**Water**	**Built-up land**	**Bare land**
**2000**	Crop land	27448.59	310.75	20.3	887.67	1244.43	16.43
	Forest land	164.36	17043.51	30.21	40.52	222.8	1.04
	Grassland	9.11	51.66	1336.06	19.12	25.29	0.93
	Water	260.69	20.16	6.3	5283.14	127.2	38.51
	Built-up land	85	11.57	1.53	32.86	2939.69	2
	Bare land	10.78	1.26	1.76	103.44	9.55	120.32
